# Protocol for a feasibility cluster randomised controlled trial of a peer-led school-based intervention to increase the physical activity of adolescent girls (PLAN-A)

**DOI:** 10.1186/s40814-015-0045-8

**Published:** 2016-01-15

**Authors:** Simon J. Sebire, Mark J. Edwards, Rona Campbell, Russell Jago, Ruth Kipping, Kathryn Banfield, Keeley Tomkinson, Kirsty Garfield, Ronan A. Lyons, Joanne Simon, Peter S. Blair, William Hollingworth

**Affiliations:** 1 Centre for Exercise, Nutrition & Health Sciences, School for Policy Studies, University of Bristol, Bristol, UK; 2 School of Social & Community Medicine, University of Bristol, Bristol, UK; 3 Farr Institute, Swansea University Medical School, Swansea, UK

**Keywords:** Physical activity, Peers, Adolescent girls, Intervention, School

## Abstract

**Background:**

Physical activity levels are low amongst adolescent girls, and this population faces specific barriers to being active. Peer influences on health behaviours are important in adolescence and peer-led interventions might hold promise to change behaviour. This paper describes the protocol for a feasibility cluster randomised controlled trial of Peer-Led physical Activity iNtervention for Adolescent girls (PLAN-A), a peer-led intervention aimed at increasing adolescent girls’ physical activity levels.

**Methods/design:**

A two-arm cluster randomised feasibility trial will be conducted in six secondary schools (intervention *n* = 4; control *n* = 2) with year 8 (12–13 years old) girls. The intervention will operate at a year group level and consist of year 8 girls nominating influential peers within their year group to become peer-supporters. Approximately 15 % of the cohort will receive 3 days of training about physical activity and interpersonal communication skills. Peer-supporters will then informally diffuse messages about physical activity amongst their friends for 10 weeks. Data will be collected at baseline (time 0 (T0)), immediately after the intervention (time 1 (T1)) and 12 months after baseline measures (time 2 (T2)). In this feasibility trial, the primary interest is in the recruitment of schools and participants (both year 8 girls and peer-supporters), delivery and receipt of the intervention, data provision rates and identifying the cost categories for future economic analysis. Physical activity will be assessed using 7-day accelerometry, with the likely primary outcome in a fully-powered trial being daily minutes of moderate-to-vigorous physical activity. Participants will also complete psychosocial questionnaires at each time point: assessing motivation, self-esteem and peer physical activity norms. Data analysis will be largely descriptive and focus on recruitment, attendance and data provision rates. The findings will inform the sample size required for a definitive trial. A detailed process evaluation using qualitative and quantitative methods will be conducted with a variety of stakeholders (i.e. pupils, parents, teachers and peer-supporter trainers) to identify areas of success and necessary improvements prior to proceeding to a definitive trial.

**Discussion:**

This paper describes the protocol for the PLAN-A feasibility cluster randomised controlled trial which will provide the information necessary to design a fully-powered trial should PLAN-A demonstrate evidence of promise.

**Trial Registration:**

ISRCTN12543546

## Background

Amongst children and adolescents, physical activity (PA) is associated with lower levels of cholesterol and blood lipids and favourable blood pressure and body composition [1]. There is also evidence that PA is associated with young people’s mental health [2] and academic performance [3]. Despite the benefits of being physically active during the early years, PA levels decline during childhood [4]. Throughout childhood and adolescence, girls are less active than boys [5] and the age-related decline in PA (particularly from early adolescence) is steeper for girls. In England, when measured objectively, 96 % of girls aged 11–15 years performed less than 30 min of moderate-to-vigorous PA (MVPA) per day and none met government recommendations of 60 min of MVPA per day [6].

The psychological correlates of adolescent girls’ PA participation include enjoyment, perceived competence, self-efficacy and physical self-perceptions [7]. Further, qualitative research suggests that these factors are intertwined with girls’ social (often school) context and changes to friendship groups, peer support, perceived competence, competing priorities, self-presentational concerns and ‘sporty’ gender stereotypes experienced during the transition from primary to secondary school which may also contribute to the observed decline in girls’ PA [8–10]. PA interventions aimed at girls have produced small but significant positive effects and larger effects have been observed for interventions that targeted girls only and used educational and multi-component designs [11]. As such, while it may be possible to increase girls’ PA, new and more effective interventions are needed.

Promoting young people’s health in schools is a public health priority [12], and school-based interventions can reach many young people over a sustained period. However, a 2009 Cochrane review of school-based PA interventions showed that none were conducted in the UK that involved adolescents and the non-UK interventions for adolescents did not increase PA [13]. Current school-based interventions have focussed on ‘top-down approaches of providing education and short-term structured PA’ [13], and there is a need for alternative designs. One approach is to develop interventions which capitalise on naturally occurring determinants within target populations and sustainable health promotion mechanisms (i.e. peer groups and their influence on PA) to promote long-term PA behaviour.

Peers play a central role in influencing adolescents’ PA and its determinants through providing peer support, co-participation in PA, peer norms, friendship quality, peer affiliation and peer victimisation [14]. This is supported by social network research which has shown that adolescents choose friends who are similarly active and that they may alter their PA over time to be more like their friends’ [15]. This work highlights the potential promise of interventions, especially amongst girls, to increase PA which capitalise on existing peer processes in schools by promoting peer support and enhancing peer communication skills [14].

Peer-led interventions have targeted a range of health behaviours amongst young people, including smoking, asthma, alcohol consumption, drug use, PA and sedentary behaviour [16–18]. A recent review of ten peer-led PA interventions [18] found that only two targeted young people: one small special population [19] and one insufficiently-reported study [20], and neither was conducted in the UK. Overall, consistent positive effects of the interventions on PA behaviour were reported, suggesting that peer-led interventions are viable; however, the existing research is limited by a lack of high quality controlled trials amongst adolescents, interventions which are largely atheoretical and a sole focus on formal methods of peer-peer delivery (e.g. leading educational classes, organised co-participation and formal advice giving) which are both time limited and intensive.

An alternative peer-led approach is to train peer-supporters to informally diffuse health promotion messages to their peers. This approach is based on Diffusion of Innovations theory [21] which conceptualises how ideas, beliefs or behaviours are informally communicated through members of a social system. This theory was adopted in the ASSIST (A Stop Smoking in Schools Trial) study [16]. The ASSIST intervention involves asking pupils within a school year group to nominate influential peers. The nominated individuals then receive training to informally diffuse messages to their peers about the target health behaviour (e.g. not smoking) for 10 weeks. In a cluster randomised controlled trial of ASSIST, which comprised 10,730 school children aged 12–13 years from England and Wales, those who received the intervention had lower odds of being a smoker compared to pupils in the control condition immediately after the intervention (OR = 0.75, 95 % CI = 0.55 to 1.01) and at 1 (OR = 0.77, 95 % CI = 0.59 to 0.99)- and 2 (OR = 0.85, 95 % CI = 0.72 to 1.02)-year follow-up [16].

The ASSIST model was adopted in an exploratory trial of the Activity and Healthy Eating in ADolecence (AHEAD) project, an obesity prevention intervention which targeted the diffusion of PA and healthy eating messages amongst 12- to 13-year-olds [22]. Although feasible to implement and well received, the intervention did not show evidence of promise to increase healthy eating or PA. It was concluded that targeting two behaviours was too complex and the healthy eating component was resource intensive and costly [22]. The results of ASSIST and AHEAD suggest that while informal school-based peer-led interventions can be effective in changing young people’s health behaviours, behaviour change messages intended for peer-diffusion need to be simple and may benefit from targeting more specific behavioural determinants of particular populations. No previous studies have used the Diffusion of Innovations approach to specifically increase PA levels of adolescent girls [21].

Interventions which target theoretical mechanisms of behaviour change are likely to be more effective than those that do not [23], yet few peer-led PA interventions incorporate theoretical principles [18]. Diffusion of Innovations theory remains a fundamental framework for harnessing the influential capacities of *change agents* (e.g. year 8 girls identified as opinion leaders by their peers); however, it does not help guide either the content or the behaviour change techniques adopted in the peer-training intervention. Self-determination theory (SDT) [24] is a framework which concerns the personal and social conditions needed to foster high quality motivation for behaviour change and has been used to understand motivation for PA amongst children and adolescents [10, 25, 26] and guide PA interventions [27], including a peer-led PA intervention for older adults [28]. SDT contends that autonomous motivation for PA (based on authentic choices, inherent satisfaction or personal value) is associated with positive behavioural, affective and cognitive outcomes, whereas controlled motivation (based on guilt or compliance with others’ demands) undermines these outcomes. Autonomous motivation is supported by the degree to which the social environment satisfies, and individuals perceive the satisfaction of three psychological needs: autonomy, competence and social belonging. Research amongst children, adolescents and adults has identified positive associations between autonomous versus controlled motivation and PA [26, 29], positive affect, challenge-seeking [29] and quality of life [30]. SDT is well suited to a peer-led intervention model because peer-supporters can create a social climate that can either undermine or facilitate their friends’ interest in PA [18]. It is also possible that peer-supporters could create a social environment which is supportive of other antecedents to autonomous motivation including health and affiliation motives, perceptions of competence, connectedness and social support and realistic choices and options of how to be physically active [8, 10].

### Study objectives

The aim of this study is to assess the feasibility of the ‘PLAN-A’ (Peer-Led physical Activity iNtervention for Adolescent girls) peer-led PA intervention, adapted from the ASSIST model, which is designed to increase the PA of adolescent girls. In a definitive trial, the primary research question would test the effectiveness of the PLAN-A intervention in increasing the PA levels of year 8 girls. The aims of this feasibility study are to:

1. Estimate the recruitment rate of year 8 girls as peer-supporters and non-peer-supporters and monitor their attendance at peer-supporter training.
2. Qualitatively examine the acceptability of the intervention to pupils, peer-supporter trainers, schools and parents and identify necessary refinements.
3. Estimate accelerometer and questionnaire data provision rates, examine data quality and explore the implications of missing accelerometer data in terms of how these data might be imputed in a definitive trial.
4. Estimate the potential effect of the intervention on daily accelerometer-derived MVPA and secondary PA-related and psychological variables immediately after the intervention (time 1 (T1)) and at 12 months after baseline (time 2 (T2)).
5. Estimate the school-related intraclass correlation coefficient (ICC) for daily MVPA, combining data from this project with our data from other local secondary schools.
6. Estimate the sample size for an adequately powered definitive trial evaluation.
7. Identify and test the feasibility of collecting the data needed to cost the intervention and conduct a cost-effectiveness analysis in a definitive trial.
8. Examine the consent rate (participants and data custodians) for data linkage to academic achievement, attendance and health records.

## Methods/design

**Fig. 1:**
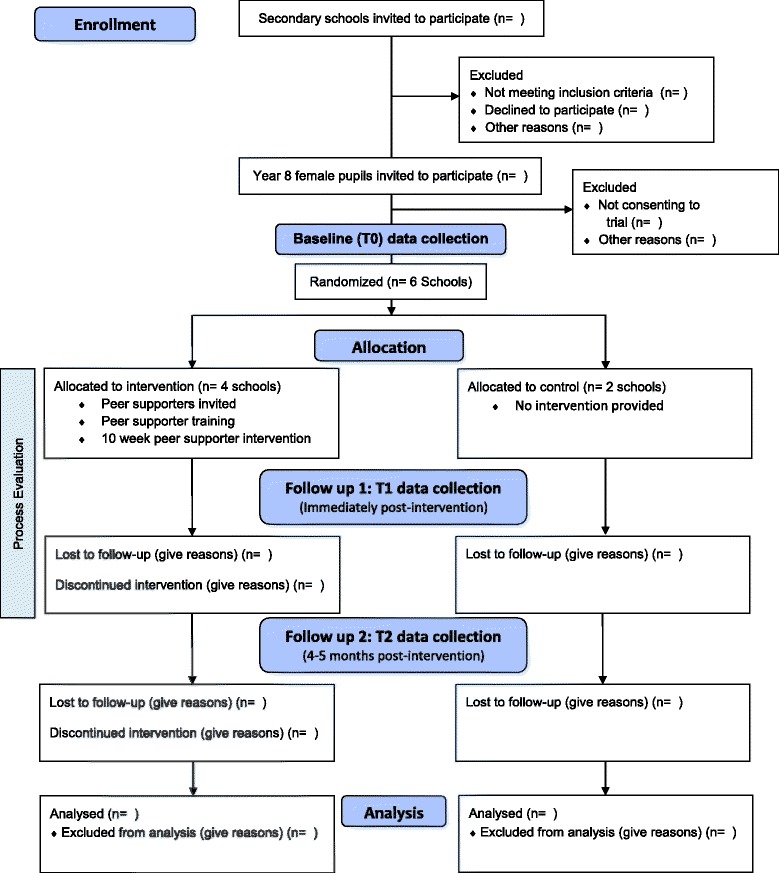
Flow diagram of the PLAN-A feasibility trial

The design is a two-arm cluster randomised controlled trial comparing the PLAN-A intervention against a usual-practice control conducted in six secondary schools, of which four schools will receive the intervention and two will serve as controls. Data will be collected at three time points: baseline (time 0 (T0)), immediately after the 10 week intervention (T1) and 12 months post-baseline (T2, 5–6 months post-intervention). Figure 1 shows the study flow diagram. The project has been approved by the research ethics committee of the School for Policy Studies at the University of Bristol (Ref: SPSREC14-15.A27).

### Setting

Eligible settings will be state-maintained secondary schools in Wiltshire and South Gloucestershire, in South West England, which are above the median of the local Pupil Premium Indicator (i.e. more deprived) (https://www.gov.uk/government/publications/pupil-premium-2015-to-2016-allocations). Special educational needs schools will be excluded. The feasibility trial will be conducted in six secondary schools, three from each area.

### Recruitment

#### School recruitment

The ASSIST programme is delivered in some schools in the study areas, and it would not be possible to run ASSIST and PLAN-A concurrently within the context of a randomised controlled trial (RCT). As such, the study team will work with Wiltshire and South Gloucestershire collaborators to identify schools where ASSIST is not delivered. The remaining schools will be invited to participate via a letter and follow up emails to the respective head teachers and other relevant school staff. Schools which express interest will be contacted and provided with further information. Reserve schools will be recruited to allow for school withdrawal prior to baseline data collection.

#### Student recruitment

In participating schools, a presentation will be made to all girls in year 8 (in the first term of year 8) to inform them about the trial. This will include details on the intervention, control conditions and the chance of the school being in either condition. All girls will be given study information for themselves and their parents. All year 8 girls in participating schools will be eligible to take part in the study.

### Consent

As the intervention operates at a year group level, consent for pupils to participate in the trial will be sought through parent opt-out consent. Parent and child information sheets and opt-out forms will be distributed to all year 8 girls (in an envelope addressed to ‘Parents’) at the point of recruitment and need to be returned prior to baseline data collection. Parents of girls invited to be peer-supporters will be asked to provide written informed consent to allow their daughter to participate in the peer-supporter training. Peer-supporters will also be asked to assent to this role. Adult participants (e.g. peer-supporter trainers, school contacts and parents) will be asked to provide written informed consent if selected for a post-intervention process evaluation interview. Parents of study pupils and pupils themselves will be asked to consent to a hypothetical data linkage scenario separately to consenting to the trial (i.e. data linkage will not be performed, but we will examine the consent rate). Data custodians (i.e. schools or Local Authorities (LAs)) will also be asked to consent to a hypothetical data linkage scenario to assess the feasibility of linking pupils’ study identification number to educational attainment, attendance and health data held by LAs as an efficient means of obtaining such data.

### Allocation strategy

School is the unit of randomisation. Six schools will be randomly allocated, stratified by LA area (Wiltshire vs. South Gloucestershire), after baseline data collection has been completed, at an intervention to control ratio of 2:1 within LA area. Four schools will be allocated to the intervention arm and two schools allocated to the control arm. Allocation will be performed (computer generated allocation) by an independent member of the Bristol Randomised Trials Collaboration who will be blind to the school identity. The statistician and all team members apart from the Project Manager, Research Assistants and Fieldworkers will be blind to the allocation of schools to trial arms.

### The PLAN-A intervention

The PLAN-A intervention is based on the ASSIST [16] model and involves the following components: (a) train-the-trainers programme, (b) peer-nomination, (c) peer-supporter training and (d) a 10-week informal health message peer-diffusion period. These elements are described below.

#### Peer nomination

All year 8 girls who have not been opted-out of the study in the six schools will be asked to complete a peer nomination questionnaire (as used in ASSIST), in which they identify the female peers they perceive to be influential in their year at school. Nomination will take place before randomisation of schools, and the girls who are nominated in what later become the intervention schools will be alerted to this soon after randomisation. The highest scoring 18 % (those with most nominations) within intervention schools will be invited to be peer-supporters. These girls will attend an in-school recruitment meeting with a study staff member. The meeting will outline the peer-supporter role, and pupil and parent information sheets and consent forms will be handed out. Pupils can opt-out of being a peer-supporter at this point. It is expected that ≥15 % of the year group take on the peer-supporter role, as outlined in Diffusion of Innovations theory [21].

#### Train-the-trainers programme

The girls who consent to the peer-supporter role will receive peer-supporter training (see below). This will be delivered by two peer-supporter trainers who will have attended a 3-day (≈15 h) education programme delivered by the study team. Peer-supporter trainers will be individuals who have either health promotion/PA knowledge (e.g. members of LA Healthy Lifestyles teams) and/or experience of facilitating group work with young people (e.g. youth workers, theatre company members). Training will cover the background to the project, fundamentals of Diffusion of Innovations and SDT, key information about PA and all session plans and activities. Training will be practical and provide opportunities to rehearse session delivery. Trainers will be provided with an intervention manual which will include all the logistical details, session plans and resources needed to deliver the training. Trainers will be reimbursed at an hourly rate to attend the train-the-trainers programme and deliver the peer-supporter training.

#### Peer-supporter training

Peer-supporters will attend a 2-day course to develop their skills, knowledge and confidence to promote PA amongst peers. Training will be held off-site and led by two external peer-supporter trainers who will have attended the train-the-trainers programme. The peer-supporter training will build on transferable activities from the previously used ASSIST and AHEAD training and include content focused on PA and interpersonal skills and personal qualities needed to be a peer-supporter. The key components of the peer-supporter training are shown in Table 1 alongside how the intervention components map on to behaviour change techniques [31] and SDT constructs. The content will be informed by SDT and will aim to build the girls’ perceived autonomy, competence and sense of social support for being a peer-supporter and for PA. Peer-supporters will be encouraged to keep these concepts in mind when having informal conversations with their peers. At the approximate mid-point of the 10-week intervention period (see below), peer-supporters will attend a 1-day top-up training event which will follow a similar format to the first 2 days and focus on sharing experiences, problem solving and reinforcing key messages about PA and peer-supporting. Lunch and refreshments will be provided at all training days. Training will take place on school days.

**Table 1:** PLAN-A peer-supporter training intervention components, behaviour change techniques and behavioural mediators targeted

Training session/activity/tasks	Behaviour change technique [31]	Behavioural mediators
Physical activity content		
Physical activity knowledge: examining pre-existing knowledge, exploring PA myths, interactive tasks to find out what counts as PA, PA recommendations and levels of PA in adolescent girls.	• Provide information on consequences of behaviour in general • Provide normative information on others’ behaviour • Goal setting (outcome)	Knowledge, competence
Fitting physical activity in: peer-supporters analyse ‘a day in their life’, identify existing PA and sedentary time and places and means by which to add in PA. Working with others to support them to identify how to fit PA into daily life and practical activities to reduce sedentary time in a range of situations.	• Barrier identification/problem solving • Provide information on where and when to perform the behaviour • Prompting focus on past success • Prompting generalisation of a target behaviour • Plan social support/social change • Time management	Autonomy, competence, relatedness
Busting barriers: identification and discussion of barriers adolescent girls face to being active. Problem solving tasks to ‘bust’ the barriers.	• Barrier identification/problem solving • Prompting focus on past success • Plan social support/social change • Prompt identification as role model/position advocate	Autonomy, competence, relatedness
Confidence and competence: watching a short video about girls’ physical activity stereotypes, discussing beliefs in groups and empowering through focussing on self-esteem and past success	• Barrier identification/problem solving • Prompting focus on past success	Autonomy, competence, relatedness, self-esteem
Goal setting: learning how to set ‘SMART goals’ and planning two peer-supporter/activity goals.	• Goal setting (outcome) • Action planning • Prompt identification as role model/position advocate	Autonomy, competence
Peer-supporter content		
Identifying personal peer-supporter attributes: self-reflection on personal skills and interests which may make them a good peer-supporter.	• Prompt identification as role model/position advocate	Competence, self-esteem
Identifying peer-supporter skills: group work to develop a list and then pyramid of the most important peer-supporter skills.	• Plan social support/social change • Prompt identification as role model/position advocate	Competence, autonomy
When, where, who? Activity to identify the timing, situations and social circumstances in which to give peer-support.	• Action planning • Provide information on where and when to perform the behaviour • Plan social support/social change	Competence, relatedness
Listening skills: interactive games to highlight key skills related to listening to peers about being active.	• Plan social support/social change • General communication skills training	Competence, relatedness
Communication skills: written and practical role play activities building awareness of communication skills and practicing their use.	• Provide rewards contingent on successful behaviour • Prompt identification as role model/position advocate • General communication skills training	Competence, relatedness
Peer-supporter role play: reinforcement of key physical activity information/learning and combination with simple role plays using sentence starters (e.g. ‘I was at this training the other day…’	• Plan social support/social change • General communication skills training • Prompt identification as role model/position advocate	Autonomy, competence, relatedness

#### 10-week informal health message peer-diffusion period

Following the training, peer-supporters will be asked to informally promote messages about increasing PA amongst their peers for 10 weeks. Peer-supporters can support anyone they choose, although encouragement will be given in the training for peer-supporters to focus their support on girls in their year group. Peer-supporters will be given a diary to record the nature of the conversations that they have with their peers about PA. During the intervention period, peer-supporters will be able to refer to the study website for tips and email questions that they have to a dedicated study email address which will be answered and shared, anonymously, on the website as Frequently Asked Questions to help other peer-supporters.

### Control group provision

Two schools will be randomly assigned to the control arm after baseline (T0) data collection and will not receive the PLAN-A intervention. These schools will continue with their normal practice. Year 8 pupils in control schools will participate in data collection at T0, T1 and T2.

### School and student appreciation

All participating schools (intervention and control) will receive a £500 donation and a summary of the research findings at the end of the project in recognition of the time devoted to accommodating the study. To maximise timely return of accelerometers and in recognition of the time given to each data collection, all participants will receive a voucher (T0 £5, T1 £10, and T2 £10).

### Measures

#### Recruitment, retention and attendance

Key outcomes are recruitment of schools, pupils, peer-supporters and peer-supporter trainers. Recruitment rates of schools and peer-supporter trainers will be recorded. Opt-out consent and assent rates of year 8 girls to participate in the trial will be recorded. We will record attendance of the invited peer-supporters to the recruitment meeting, rate of consent (parents) and assent (pupils) to the peer-supporter role and attendance at the peer-supporter training and top-up training.

#### Outcome measures

All measures will be taken at baseline (T0), immediately after the intervention period (T1) and 12 months after baseline (T2). The likely primary outcome in a definitive trial would be weekday minutes of MVPA per day, measured by accelerometry. This method can provide reliable estimates of young people’s PA and is validated amongst young people [32]. Participants will be asked to wear an ActiGraph wGT3X-BT accelerometer for 7 days at T0, T1 and T2. Periods of ≥60 min of zero counts will be recorded as non-wear and removed. Participants will be included in analysis if they provide at least 2 days of valid weekday data (i.e. 500 min of data between 05:00 and 23:59). Mean minutes of daily MVPA will be estimated using the Evenson [33] cut-point which has been found to be the most accurate threshold for adolescents [34]. The intervention could reduce the amount of time participants spend sedentary and/or their mode of travel to school (i.e. active vs. passive). As such, in addition to exploring other potential secondary outcomes (e.g. minutes of MVPA on weekend days and the proportion of girls meeting government physical activity recommendations), we will estimate participants’ sedentary time using accelerometery based on a cut-point of less than 100 cpm [33]. To provide an indication of change in sedentary behaviours, we will also assess pupil self-reported screen-viewing at T0, T1 and T2 [35]. Participants will also report their usual travel mode to and from school at each time point.

At all time points, the following psychosocial variables will be assessed by self-report questionnaire: autonomous and controlled motivation for PA [36], autonomy [29], competence [37] and relatedness [29] need satisfaction in PA, self-esteem [38], PA self-efficacy [39], PA social support from friends [40] and PA peer norms [41]. These may form secondary outcomes or mediators in a definitive trial. At each time point, pupils will also self-report two items developed for this study to assess year-group-based peer support for PA: ‘Has anyone in your year group talked with you recently about physical activity?’ (Yes, No, I’m not sure) and ‘Did talking to any one in your year help you to be more active?’ (Yes, No, I’m not sure, I didn’t speak to anyone). The EQ-5D-Y [42] will be used to measure self-reported quality of life at each time point. At T0 only, participants will complete additional questionnaire items to report demographic information.

### Process evaluation

A detailed process evaluation will examine acceptability of the intervention and methods, intervention delivery and implementation, potential intervention mechanisms and the influence of school context [43] using qualitative and quantitative approaches amongst peer-supporters, non-peer-supporter pupils, peer-supporter trainers, parents of peer-supporters and school contacts. The process evaluation methods are shown in Table 2.

**Table 2:** Summary of the PLAN-A feasibility trial process evaluation

Participant	Method	Information gathered
Peer-supporters	Post-training questionnaire (All peer-supporters in 4 schools)	Open ended questions regarding enjoyment, identifying memorable facts, ratings of confidence to peer support and anticipated difficulties. Quantitative ratings of enjoyment of training, duration, confidence, venue and trainers (including trainer autonomy support [48])
	Post-intervention focus groups (1 per school, *n* ≈ 30)	Perceptions of training and intervention: peer-supporter recruitment, training content, logistics and trainer, being a peer-supporter (e.g. successes and challenges, use of the email/web support, interpersonal and environmental barriers/facilitators)
Non-peer-supporter pupils	T0, T1 and T2 questionnaire	Two items assessing perceived contact/conversations with year 8 pupils about physical activity
	Post-intervention focus groups (1 per school, *n* ≈ 30)	Perceptions of receiving peer-support, awareness of peer-supporters, peer nomination, perceptions of impact, research methods.
Peer-supporter trainers	Post-training questionnaire (*n* ≈ 4)	Attendance, absences and reasons for absence, *N* pupils who did not complete training and reasons, training arrangements, degree to which training goals were met, pupil engagement and response to training
	Post-delivery semi-structured interviews (*n* ≈ 4)	Perceptions of train-the-trainers programme and 2-day peer-supporter training, resources, venue, successes, challenges and refinements
	Training observation	Degree to which lesson plans were delivered as manualised. Qualitative observations of successes and challenges.
Parents of peer-supporters	Semi-structured interviews (*n* ≈ 12)	Awareness of the intervention, views on acceptability of training, intervention, influence of family context and study child’s activity and attitudes. Perception of impact.
School contact	Semi-structured interviews (*n* = 6)	Peer nomination, training, intervention, difficulties and successes. Acceptability of research methods.

School context

School level data: school and year 8 size, pupil premium allocation

School contact questionnaires: assessment of school PA provisions, school policies, PA in the curriculum and school attitude towards PA

#### Economic data

Data will be collected to estimate intervention costs and examine the feasibility of calculating cost-effectiveness analysis alongside a definitive trial. Resource use data, including intervention materials, venue costs, staff, trainer and pupil time, expenses, travel and administration costs, will be collected prospectively during each stage of the intervention using expense claim forms and data collection forms completed by the research team and school contact.

### Sample size

As the study is a feasibility trial, a formal power calculation based on detecting evidence for effectiveness has not been conducted. The feasibility RCT will be conducted in six schools (four interventions, two controls). Based on recent experience in local secondary schools, we anticipate that there will be between 60 and 100 female pupils in year 8 per school. Therefore, we expect that the sample size will range between 360 and 600 girls, including between 36 and 60 peer-supporters from the four intervention schools. This is a pragmatically chosen sample to allow us to identify evidence of feasibility, recruitment rates and any problems with the intervention or research methods. An aim of the feasibility study is to calculate the required sample size for a definitive cluster RCT evaluation of the PLAN-A intervention. As such, we will estimate the variability in the likely definitive trial outcome (minutes of MVPA per day) using an ICC. Commensurate with recent recommendations [44] and as the ICC for MVPA in the study will be derived only from six schools, to obtain a more accurate ICC estimate for inclusion in a power calculation for a definitive trial, the ICC will be informally compared to those obtained in our previous research which has used the same measures amongst adolescent girls (e.g. Bristol Girls’ Dance Project) [45].

### Data analysis

#### Quantitative analysis

The main outcomes in this feasibility trial are the consent and recruitment rates (year 8 cohorts and peer-supporters), number and proportion of peer-supporters who receive training, and data provision rates. Analysis of these data will be mainly descriptive (means and SD, or N and %) as appropriate. Descriptive comparisons of these data will be made between intervention and control arms. Loss to follow-up in intervention and control groups will be reported. The implications of missing accelerometer data will be investigated using multiple imputations. Demographic characteristics will be summarised descriptively as appropriate.

Evidence of promise (i.e. whether the intervention could lead to an increase in daily MVPA) will be examined using appropriate multivariable linear regression models to compare between group differences in means and 95 % CIs of MVPA, adjusted for baseline PA and clustering of participants within schools. As the study is not powered to detect effectiveness, *p* values will not be considered and the focus will be on whether the 95 % CIs include a meaningful difference in MVPA. Based on previous research [46], sample sizes for a future definitive trial will be based on detecting a 10-min per day difference in MVPA between trial arms, estimated using the ICC for daily MVPA and based on combinations of different alpha (i.e. significance level) and beta (i.e. power) rates.

A public sector perspective will be taken in the economic analysis. Where available, national unit costs for staff time will be used to increase the generalisability of findings. Cost per pupil will be estimated by dividing the costs of the peer-supporter programme at each school by the number of female participants in the school year group. We will calculate an incremental cost-effectiveness ratio (ICER) by dividing the mean cost per pupil of the intervention by the difference in daily MVPA in the intervention and control schools. The analysis is designed to explore the affordability and potential cost-effectiveness of the intervention rather than provide a definitive comparison.

#### Qualitative analysis

Recordings of interviews and focus groups will be transcribed verbatim. Thematic analysis [47] will be used to analyse these data. Transcripts will be read and reread, and initial codes that categorise the content of each transcript will be produced. These initial codes will then be iteratively refined to produce emergent themes. We will examine divergence and convergence within and between interviews and focus groups and compare the experiences of the intervention across the different participant groups to develop a comprehensive understanding of the intervention acceptability, implementation and mechanisms of impact.

Further details on the statistical and economic and qualitative analysis plans will be made available on the study website (www.plan-a-project.org).

## Discussion

The PLAN-A feasibility trial aims to use a peer-led intervention to increase the PA of 12–13-year-old girls. As PA levels are low amongst British girls and decrease from childhood to adolescence [4], new intervention approaches are needed which are tailored to the unique determinants of PA amongst adolescent girls and harness the potential power of their existing and accepted social networks. The PLAN-A intervention builds on the effective ASSIST peer-led smoking cessation intervention model and the feasibility trial aims to assess the potential of this approach to increase adolescent girls’ PA. The study will also provide the information needed to design a definitive cluster RCT should evidence of promise be shown.

### Trial status

The feasibility trial is registered with the ISRCTN (ISRCTN12543546). Ethical approval for the project was received from the Research Ethics Committee of the School for Policy Studies at the University of Bristol (Ref: SPSREC14-15.A27)). Pupil recruitment and baseline (T0) data collection are planned for September/October 2015.

## Abbreviations

AHEAD: Activity and Healthy Eating in ADolecence
ASSIST: A Stop Smoking in Schools Trial
ICC: intraclass correlation coefficient
ICER: incremental cost effectiveness ratio
LA: Local Authority
MVPA: moderate to vigorous physical activity
PA: physical activity
PLAN-A: Peer-Led physical Activity iNtervention for Adolescent girls
RCT: randomised controlled trial
SDT: self-determination theory
T0, T1, T2: time 0, time 1, time 2
